# Nitric Oxide and Reactive Oxygen Species Mediate Metabolic Changes in Barley Seed Embryo during Germination

**DOI:** 10.3389/fpls.2016.00138

**Published:** 2016-02-16

**Authors:** Zhenguo Ma, Frédéric Marsolais, Natalia V. Bykova, Abir U. Igamberdiev

**Affiliations:** ^1^Department of Biology, Memorial University of Newfoundland, St. John’sNL, Canada; ^2^Genomics and Biotechnology, London Research and Development Centre, Agriculture and Agri-Food CanadaLondon, ON, Canada; ^3^Department of Biology, University of Western OntarioLondon, ON, Canada; ^4^Morden Research and Development Centre, Agriculture and Agri-Food CanadaMorden, MB, Canada

**Keywords:** barley (*Hordeum vulgare* L.), dormancy, germination, nitric oxide, reactive nitrogen species, reactive oxygen species

## Abstract

The levels of nitric oxide (NO) and reactive oxygen species (ROS), ATP/ADP ratios, reduction levels of ascorbate and glutathione, expression of the genes encoding proteins involved in metabolism of NO and activities of the enzymes involved in fermentation and in metabolism of NO and ROS were studied in the embryos of germinating seeds of two barley (*Hordeum vulgare* L.) cultivars differing in dormancy level. The level of NO production continuously increased after imbibition while the level of nitrosylated SH-groups in proteins increased. This corresponded to the decrease of free SH-groups in proteins. At early stage of germination (0–48 h post imbibition) the genes encoding class 1 phytoglobin (the protein scavenging NO) and *S*-nitrosoglutathione reductase (scavenging *S*-nitrosoglutathione) were markedly expressed. More dormant cultivar exhibited lower ATP/ADP and ascorbate/dehydroascorbate ratios and lower lactate and alcohol dehydrogenase activities, while the production of NO and nitrosylation of proteins was higher as compared to the non-dormant cultivar. The obtained data indicate that at the onset of germination NO is actively generated causing nitrosylation of SH-groups and a switch from respiration to fermentation. After radicle protrusion the metabolism changes in a more reducing type as recorded by ratio of reduced and oxidized glutathione and ascorbate. The turnover of NO by the scavenging systems (phytoglobin, *S*-nitrosoglutathione reductase and interaction with ROS) might contribute to the maintenance of redox and energy balance of germinating seeds and lead to alleviation of dormancy.

## Introduction

The early stage of seed germination starting from the uptake of water by dry seeds is a challenge for plants, in particular because seed coat represents a robust barrier for oxygen (Bewley, 1997). Only after radicle protrusion the mobilization of storage reserves becomes not significantly limited by oxygen supply (Taylorson and Hendricks, 1977; Bewley and Black, 1994; Bewley, 1997). As a consequence, after imbibition seeds develop anaerobic conditions limiting oxidative respiration (reviewed in Bykova et al., 2015). Despite this, seed ATP levels and energy charge remain high in early germination (Duff et al., 1998; Logan et al., 2001; Benamar et al., 2003). The increase of reduction level of NAD, NADP, and other electron transferring compounds upon oxygen depletion immediately after imbibition results in the formation of reactive species of oxygen (ROS) and of nitrogen (RNS) (Bethke, 2009).

It has been suggested that generation of nitric oxide (NO) under seed coat is increased upon depletion of oxygen representing a mechanism of supporting redox and energy balance in germinating seeds together with activation of fermentation processes (Debska et al., 2013). In germination, after the initial depletion of oxygen within several hours, the transition to the mainly anaerobic step takes place, characterized by NO production. These phases correspond to the stages I and II described by Bewley and Black (1994). While the generation of NO is an unavoidable consequence of low oxygen and high reduction level of electron transferring compounds (Igamberdiev and Hill, 2004), the turnover of NO that leads to balancing of redox and energy levels is achieved via the systems participating in scavenging of RNS such as class 1 plant hemoglobin (phytoglobin; Igamberdiev et al., 2006) and S-nitrosoglutathione reductase, which detoxifies nitrosylated glutathione returning it to the active glutathione pool and represents one of the reactions catalyzed by the class III alcohol dehydrogenase (Corpas et al., 2013). This results in the oxidation of NAD(P)H and possibly in the support of ATP formation by mitochondria (Stoimenova et al., 2007). However, the particular aspects of NO and RNS metabolism in germinating seeds are not investigated in detail and need further clarification.

It has been suggested that NO and ROS are crucial for dormancy breaking, while generation of NO under seed coat is increased upon depletion of oxygen representing a mechanism of supporting redox and energy balance in germinating seeds together with the activation of fermentation processes (Bethke et al., 2004, 2007; Igamberdiev and Hill, 2004). While ROS formation starts upon imbibition, the NO production is triggered by the progressing depletion of oxygen under seed coat upon germination. NO nitrosylates proteins and glutathione, while ROS can interact with NO and facilitate its scavenging (Wulff et al., 2009). The interplay between NO and ROS is important for understanding how the seed breaks dormancy and maintains its energy status during germination (Bykova et al., 2015).

In this paper we have presented a wide-range study of the development of oxidative metabolism in embryo during the first 2 days of germination of the cereal (barley) seeds of two cultivars with different germination rates. The main part of embryo in early germination is scutellum which supplies intermediates for carbohydrate and amino acid synthesis and contributes to acidification of endosperm. We have traced the development of production of NO, ROS and their scavenging systems in the course of germination of two barley cultivars differing in dormancy level. It is concluded that NO production results in the increase of protein nitrosylation. The mechanisms involved in scavenging of NO and RNS include the expression of phytoglobin and GSNO-reductase, as well as the interaction of NO and superoxide anion with the following degradation of peroxynitrite. The role of NO turnover during the early stage of plant development is discussed in relation to the maintenance of redox and energy levels in cells, which supports the development of germinating seeds and facilitates the efficiency of responses to biotic and abiotic stress factors in the course of germination process and dormancy breaking.

## Materials and Methods

### Barley Seeds

Seeds of two barley (*Hordeum vulgare* L.) cultivars, Harrington (non-dormant, 96% of seeds germinate after 24 h) and Sundre [more dormant, its germination resistance is about 1.2 days determined by the method of Gordon (1971)] were soaked with sterile deionized water from 0 (dry seeds) to 48 h on filter paper in Petri dishes. Embryos (usually 100 mg) were isolated and ground in liquid nitrogen with mortar and pestle. The Sundre embryos analyzed from 0 to 9 h after imbibition could not be distinguished into dormant and non-dormant and therefore represented a mixture of seeds with different germination potential.

### Measurement of Protein Concentration

The method of measuring concentration of proteins was based on standard protocol of Bradford reagent (Sigma–Aldrich). Bovine serum albumin was used as a standard.

### ATP and ADP Measurement

Extraction of ATP and ADP was conducted according to Joshi et al. (1989) and Yuroff et al. (2003). The tissue powder was lysed in ice-cold 2.4 M perchloric acid (1 ml to 25 mg) for 60 min on ice, centrifuged 5 min at 20,000 ×* g*, and supernatant (0.5 ml) was neutralized by 4 M KOH. Quantification of ATP in the neutralized solution was performed by chemiluminescent analysis using the protocol of ATP Detection Kit (Thermo Fisher Scientific). Content of ADP was determined using EnzyLightTM ADP Assay Kit (EADP-100, BioAssay Systems). Fine tissue powder (25 mg) was homogenized in 200 μl of ice-cold 50 mM potassium phosphate buffer (pH 7.0) and centrifuged at 12,000 ×*g* for 5 min. Supernatant was used for ADP analysis, followed the standard protocol of the kit.

### Measurement of NO Content

Preparation of hemoglobin (Hb) solution and NO measurement were conducted according to Murphy and Noack (1994). Hb (50 mg) was dissolved in 10 ml of 50 mM Tris-HCl, pH 7.0. Sodium dithionite (Na_2_S_2_O_4_, 15 mg) was added directly into fresh Hb solution (which contained mainly MetHb) and reacted for 3 min to reduce MetHb (Fe^3+^) to Hb (Fe^2+^), which immediately became HbO_2_. The prepared HbO_2_ solution was desalted through PD-10 column (GE Healthcare Life Sciences). The final concentration of HbO_2_ was determined by spectrophotometry at 415 nm (𝜀 = 131 mM^–1^ cm^–1^), adjusted to ∼10 μM and stored in dim light.

Nitric oxide was extracted from tissue powder with 50 mM Tris-HCl buffer, pH 7.0, containing 0.6% (w/v) PVP at 4°C. Supernatant was collected after centrifugation for 10 min at 15000 ×*g*. To 0.5 ml of supernatant 12.5 μl superoxide dismutase (SOD; 4,000 U/ml) and 12.5 μl catalase (10,000 U/ml) was added and incubated for 2 min at room temperature to remove ROS. A volume of 0.475 ml of the desalted HbO_2_ solution was added and the tubes were incubated for 5 min. Absorbance at 401 and 421 nm was recorded to calculate the concentration of NO in tissue using the extinction coefficient 𝜀 = 77 mM^–1^ cm^–1^.

It is recommended to use the two methods for measuring NO (Gupta and Igamberdiev, 2013). The measurement of NO content by the hemoglobin method was in agreement with the method using chemiluminescent NO analyzer CLD 88 p (Eco-Physics, Dürnten, Switzerland; not shown). However, it was not possible to compare these two methods before radicle protrusion when NO is trapped inside the seed coat, therefore we present here the data obtained by the hemoglobin method.

### Measurement of Protein SH-Groups and *S*–Nitrosylation

Measurement of protein *S*–nitrosylation was conducted according to Jaffrey et al. (2001) by reduction of R-SNO to R-SH in the presence of ascorbate followed by the assay of free thiol groups using 5,5′–dithiol–bis (2–nitrobenzoic acid; DTNB; Riddles et al., 1983). Proteins were extracted from tissue powder with 50 mM HEPES, pH 8.0, containing 1 mM EDTA, 0.1 mM neocuproine, 0.2% (w/v) SDS and 0.5% (w/v) CHAPS. The samples were centrifuged at 4°C for 10 min at 15,000 ×*g* and proteins were precipitated from supernatant by two volumes of acetone (-20°C) overnight. After centrifuging at 4°C and 15,000 ×*g* for 10 min, protein precipitate was washed four times with chilled 70% acetone. The protein samples were re-suspended in the same volume of the extraction buffer. The clear protein solution was collected to measure the quantity of R-SNO. Ascorbate (50 μl of 100 mM solution) was added in 0.9 ml samples, the same volume of H_2_O was added to control. After incubation for 1 h at 25°C, 50 μl of 10 mM DTNB in 75 mM phosphate buffer, pH 7, was added, followed by measurement of absorbance at 412 nm against control samples. The mixture of ascorbate and DTNB in extraction buffer, and DTNB in the same buffer were set up as blank for treatment and control groups, respectively. The difference of R-SH quantity between sample and control groups was used to calculate quantity of R-SNO. Namely, the quantity of R-SH generated by ascorbate treatment corresponded to that of R-SNO in proteins. The measurement of free SH-groups in proteins was conducted without ascorbate treatment.

### Measurement of Superoxide Anion and Hydrogen Peroxide

To extract $O_{2}^{•-}$, the fine powder of fresh tissue was homogenized in 8 M KOH and centrifuged for 10 min at 15000 *g* at 4°C. The superoxide anion level was measured in supernatant by the method modified from Sun and Trumpower (2003) and Dahlgren et al. (2007) at 550 nm by reduction of cytochrome *c*. Cytochrome *c* (0.1 ml, 5 mg ml^–1^) was dissolved in 1.397 ml of 0.2 M potassium phosphate buffer, pH 8.6, and 3 μl extract solution was added finally, homogenized immediately and incubated for 15 min at 25°C. In the reference set 50 units of SOD and 100 units of catalase were added to the solution. The value of 𝜀 = 21.5 mM^–1^ cm^–1^ for the reduced cytochrome c (Fe^2+^) was used, and based on a one-to-one molar stoichiometry between $O_{2}^{•-}$ produced and cytochrome *c* molecules reduced, the actual amount of $O_{2}^{•-}$ produced was quantified.

Concentration of hydrogen peroxide was measured according to the method of Lu et al. (2009). Fine powder of isolated scutella was homogenized in 6% trichloroacetic acid (TCA) for 30 min at 4°C, centrifuged at 15000 ×*g* for 10 min, and then insoluble polyvinylpyrrolidone (PVPP; 50 mg/ml) was added. The samples were centrifuged at 15000 ×*g* for additional 3 min. The extract was diluted 1000 fold with 0.1 M carbonate buffer (pH 10.2). The preparation of reagents followed the method of Pérez and Rubio (2006) and Lu et al. (2009). Ten ml of 6.5 mM luminol and 2 ml of 3 mM CoCl_2_ in 0.1 M sodium carbonate buffer (pH 10.2) were mixed, diluted to 100 ml in the same buffer and stored for at least 1 h in dark. The solution was further diluted 10 times in the same buffer and stored at 4°C in dark overnight before use. The 40 μl sample was mixed with 10 μl of the sodium carbonate buffer and the mixture was incubated at 30°C for 15 min. Catalase (500 units) was added and incubated at the same condition as a control. Ten μl of each sample and 200 μl of reaction reagent were added into 5 ml SARSTEDT tubes to measure chemiluminescence (CL) with FB 12 Luminometer (Berthold Detection Systems GmbH, Germany). The difference of CL response between each treatment and corresponding background was considered as CL specific for H_2_O_2_ in samples. The amount of H_2_O_2_ produced per gram tissue was calculated using the standard curve.

### Expression of the Genes Encoding *S*–Nitrosoglutathione Reductase and Class 1 Phytoglobin

Primers of *ADH3* (GenBank: X12734.1), *Hb* (GenBank: U94968.1), and *Mub1* (GenBank: M60175.1) for Real-Time Polymerase Chain Reaction (RT PCR) were designed using NCBI/Primer-BLAST according to known cDNA sequence of the *ADH3* and *Hb* genes encoding correspondingly GSNOR (which is a class III alcohol dehydrogenase) and class 1 phytoglobin, where *Mub1* was set up as a reference gene. Specific primers of *ADH3*, *Hb*, and *Mub1* were: ADH3-forward: 5′ - GTCTCTCAACTGGACTTGGTG - 3′ and ADH3-reverse: 5′ - CTTAGCTTGTTCGTATTTTGCAGG - 3′; Hb forward: 5′ - ACCAACCCCAAGCTCAAGAC - 3′ and Hb reverse: 5′ - CTGCCACGCCGTATTTCAAG - 3′; and Mub1-forward: 5′ - CACCGGCAAGGTAACCAG - 3′ and Mub1-reverse: 5′ - GACATAGGTGAGTCCGCAC - 3′, respectively, which were synthesized by Eurofins. Extraction of total RNA was carried out using the RNeasy Plant Mini Kit (QIAGEN) and all of the steps followed the standard protocol of its manufacturer. Concentration of total RNA was measured using NanoDrop 1000 instrument (Thermo Fisher Scientific) and 1.5 μg RNA was loaded onto 1% agarose gel to observe the quality of the extracted RNA. RNase-free DNase I treatment of RNA followed the protocol of Ambion (Thermo Fisher Scientific). Reverse transcription of RNA followed the manufacturer’s protocol for the qScript^TM^ cDNA SuperMix (Quanta Biosciences). The single strand cDNA was used as template in the following PCR. The cDNA synthesized in this step was diluted into a series of cDNA solutions to determine efficiency of the primers. PCR of *ADH3, Hb*, and *Mub1* followed the manufacturer’s protocol for the SsoFastTM EvaGreen^®^ Supermix (Bio-Rad Laboratories): 0.5 μl 10 μM forward primer, 0.5 μl 10 μM reverse primer, 1 μl cDNA (diluted eightfold) and 5 μl SsoFast™ EvaGreen^®^ Supermix were mixed and adjusted to 10 μl using nuclease-free water. Samples were transferred to Hard-Shell 96-well plate (Bio-Rad Laboratories) and sealed tightly using a transparent membrane. No template control (NTC) was set up for each biological sample and run in duplicate. Biological replicates corresponded to independent RNA extracts, and three technical replications were run for each biological replicate. The program for the RT PCR reactions was set up as enzyme activation at 95°C for 30 s, followed by 40 cycles of 95°C for 10 s and 58°C for 30 s. PCR products were viewed by electrophoresis on 2% agarose gel. PCR was performed on a CFX96 Real-Time PCR Detection System and results analyzed with Bio-Rad CFX Manager. Data was expressed as the cycle number required for reaching a threshold fluorescence value (*C*_q_). Data was normalized to the mean *C*_q_ of the reference gene, for which variation between samples was <1. The specificity of primer pairs was confirmed by melt curve analysis in comparison with controls without template. PCR efficiency was calculated from a standard curve of *C*_q_ versus the logarithm of starting template quantity. Each assay was optimized so that efficiency ranged between 97 and 101%, with a coefficient of determination (*R*^2^) > 0.99.

Determination of *S*-nitrosoglutathione reductase (GSNOR, EC 1.2.1.46) was modified from the method of Wünsche et al. (2011). GSNOR was extracted from embryo with 50 mM Tris-HCl buffer, pH 8.0, containing 100 mM NaCl and 0.1 mM EDTA. Supernatant was collected after centrifuging for 15 min at 18,000 ×*g* and passed through Sephadex G-10 column. Fifty μl of the filtered solution was taken to measure enzymatic activity at 340 nm in final 1 ml of 50 mM Tris-HCl (pH 8.0) containing 0.4 mM GSNO and 0.2 mM NADH (𝜀 = 6.22 mM^–1^cm^–1^). The reaction was initiated by adding GSNO and change of absorbance was recorded for 3 min.

### Measurement of Ascorbate and Glutathione

Ascorbate and glutathione were extracted with 6% TCA from tissue powder. The homogenate was centrifuged at 12,000 ×*g* for 20 min at 4°C. Supernatant was used for measurement of reduced and oxidized ascorbate and glutathione. Ascorbate (ASC) and dehydroascorbate (DHA) were determined according to Kampfenkel et al. (1995), and the absorbance was recorded at 525 nm using spectrophotometer. Reduced glutathione (GSH) and oxidized glutathione (GSSG) were determined according to Zaharieva and Abadía (2003). The method is based on the reaction of DTNB with GSH forming 5-thionitrobenzoic acid (TNB) recorded at 412 nm. The oxidized glutathione (GSSG) was measured after its reduction by glutathione reductase (Sigma).

### Measurement of Enzymes Involved in Oxidative Metabolism

The extraction method was modified from Murshed et al. (2008). All extraction steps were performed on ice. Catalase, SOD, alcohol and lactate dehydrogenases were extracted from embryo with 50 mM Tris-HCl buffer, pH 8.0. The enzymes of ascorbate-glutathione cycle were extracted from fine powder of isolated embryo with 50 mM MES-KOH buffer, pH 6.5, containing 40 mM KCl, 2 mM CaCl_2_, 1 mM ascorbate (for ascorbate peroxidase, added freshly). The ratio of extraction buffer to the scutellum tissue was 1 ml to 20 mg powder of tissue in fresh weight (FW). The samples were vortexed and then centrifuged at 15000 g for 10 min. Supernatant was collected as a crude enzyme solution.

Measurement of SOD (EC 1.15.1.1) was performed according to Kuthan et al. (1986) and Gupta et al. (1993) by inhibition of reduction of cytochrome *c*. SOD was extracted by 50 mM potassium phosphate buffer, pH 7, containing 0.1 mM EDTA and 1% (w/v) polyvinylpolypyrrolidone (PVP). The spectrophotometric buffer contained 0.05 M potassium phosphate (pH 7.8), 0.1 mM EDTA, and 0.05 mM xanthine. Fifty μl of crude SOD solution and 50 μl 5 mg/ml cytochrome *c* on ice were mixed with 0.85 ml of the buffer until absorbance at 550 nm was constant and finally 0.05 ml of xanthine oxidase (0.02 U/ml) was added and mixed. The increase of absorbance at 550 nm was recorded for 2 min. KCN (2 mM) was added to SOD extract to measure activity of MnSOD. The activity of Cu/Zn SOD was calculated as a difference of total SOD and MnSOD. The activity of Fe SOD, which is mainly chloroplastic, was negligible and not considered.

The methods for measuring activities of the ascorbate-glutathione cycle enzymes were modified from Nakano and Asada (1987). The assay medium for ascorbate peroxidase (APX, EC 1.11.1.11) was 50 mM potassium phosphate buffer (pH 7.0) containing 0.25 mM sodium ascorbate and 50 μl sample extract. The reaction was started by adding H_2_O_2_ (final concentration 0.25 mM) and the reaction rate was determined spectrophotometrically by absorbance change at 290 nm (coefficient of absorbance, 𝜀 = 2.8 mM^–1^ cm^–1^). Dehydroascorbate reductase (DHAR, EC 1.8.5.1) activity was measured at 265 nm (𝜀 = 14 mM^–1^ cm^–1^). The assay buffer contained 50 mM HEPES buffer (pH 7.0), 0.1 mM EDTA, 2.5 mM GSH, and 50 μl of extract. The reaction was initiated by adding freshly prepared DHA (final concentration of 0.8 mM). Monodehydroascorbate reductase (MHAR, EC 1.6.5.4) activity was measured in 50 mM HEPES buffer (pH 7.6) containing 2.5 mM ascorbate, 0.25 mM NADH, and 50 μl of the extract. The assay was initiated by adding 0.4 U cm-3 of ascorbate oxidase and the reaction rate was monitored at 340 nm (𝜀 = 6.22 mM^–1^ cm^–1^). Glutathione reductase (GR, EC 1.8.1.7) activity was measured in 50 mM HEPES buffer (pH 8.0) containing 0.5 mM EDTA, 0.25 mM NADPH, and 50 μl extract. The reaction was started by adding GSSG to final concentration of 1 mM. Catalase (EC 1.11.1.6) activity was measured at 240 nm according to Aebi (1974). In the above reactions, the change of absorbance was recorded for 3 min by Biochrom Ultrospec 4300 spectrophotometer (Amersham, UK).

Alcohol dehydrogenase (ADH; EC 1.1.1.1) was measured according Blandino et al. (1997) with ethanol and NAD^+^. Determination of lactate dehydrogenase (LDH; EC 1.1.1.27) activity was performed according to Hoffman et al. (1986) with pyruvate and NADH. Both activities were measured spectrophotometrically at 340 nm.

### Statistical Analysis

All the experiments were repeated at least three times. The data in the text, tables, and figures are expressed as means ± SD of three replicates. The differences with *P* ≤ 0.05 were considered as statistically significant.

## Results

### Germination of Barley Seeds

**FIGURE 1:**
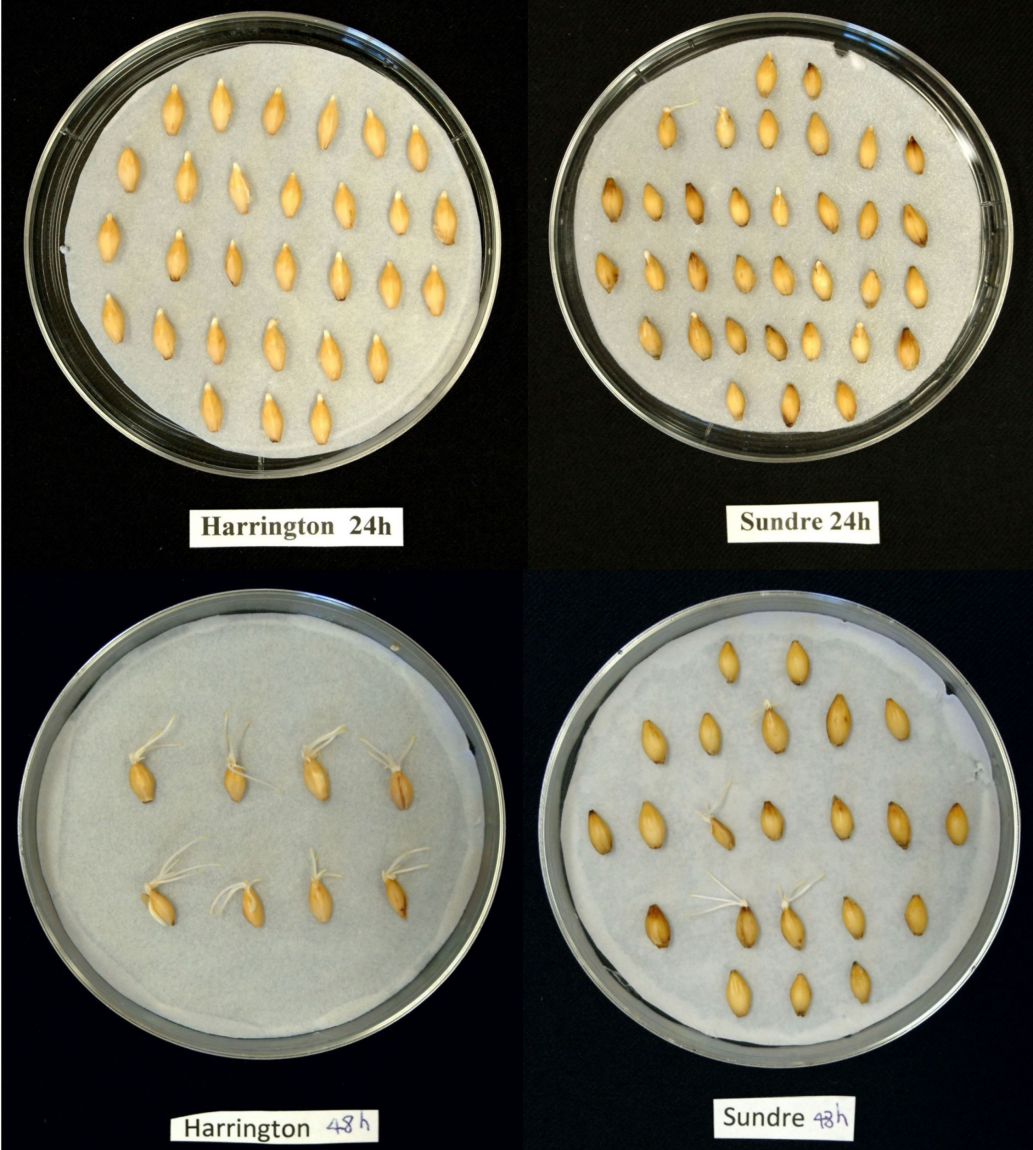
**Photos of germinating seeds of Harrington and Sundre cultivars of barley taken at 24 and 48 h post imbibition**.

The seeds of Harrington cultivar were non-dormant with the germination rate 96%. The seeds of Sundre cultivar kept the moderate level of dormancy with germination resistance 1.2 days. Practically all seeds were viable, the treatment with gibberellic acid resulted in alleviation of dormancy and germination rate of 96% for both cultivars (not shown). Therefore, the non-germinated seeds in figures represented viable seeds that kept dormancy at the particular time of assay. Radicle protrusion took place between 10 and 15 h post imbibition. **Figure 1** shows the germination of two cultivars as observed at 24 h after imbibition (after radicle protrusion in non-dormant seeds) and at 48 h (when all non-dormant seeds were germinated).

### Protein Change, ATP Level and ATP/ADP Ratio

**FIGURE 2:**
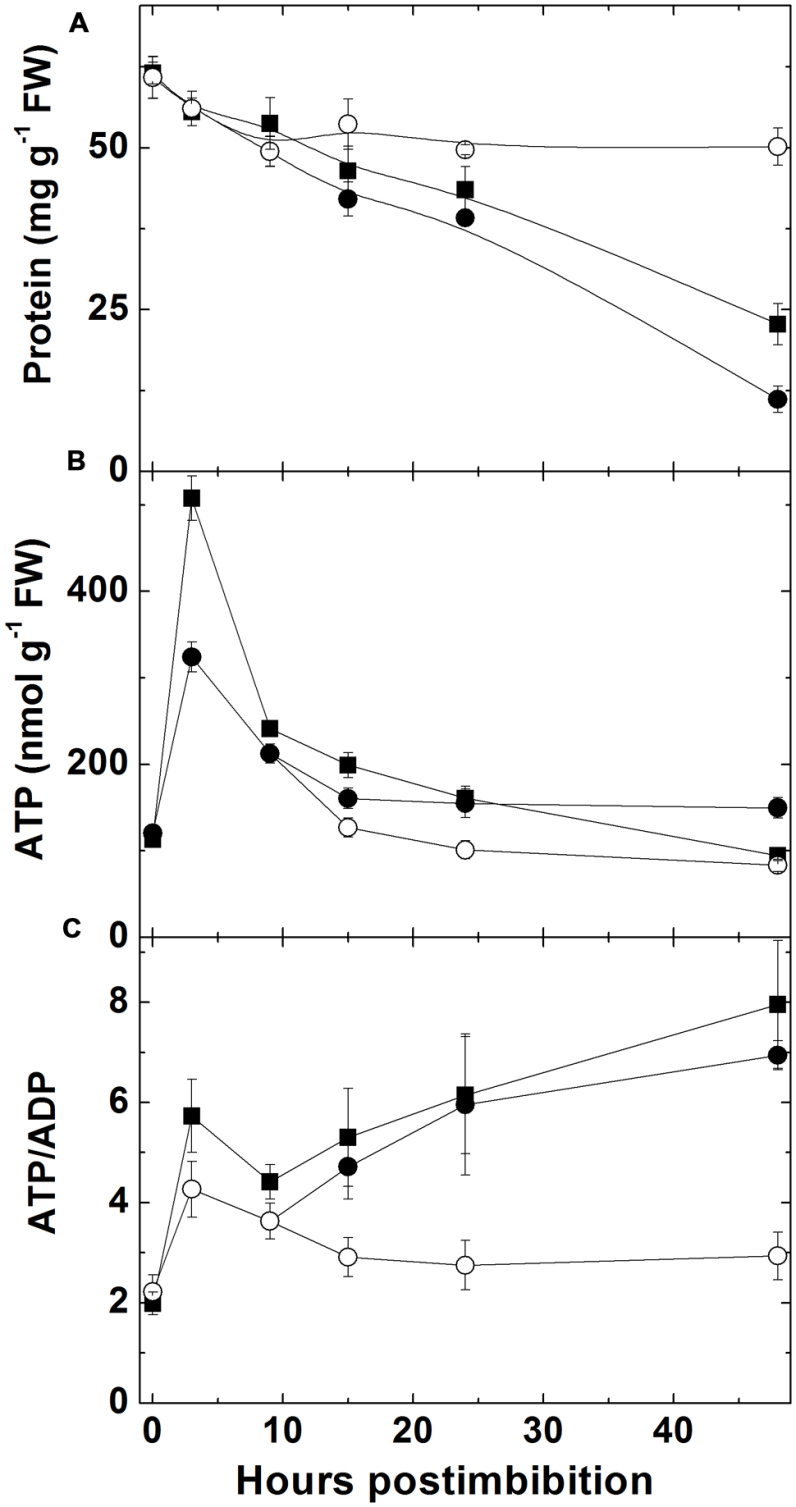
**Changes in total protein content **(A)**, ATP **(B)**, and ATP/ADP ratio **(C)** in embryo of two barley cultivars during germination.** Closed squares – Harrington cultivar, closed circles – Sundre cultivar, open circles designate non-germinated seeds of Sundre.

The total protein content in embryo (**Figure 2A**) started to decrease immediately after imbibition. The protein content decreased almost twofold during the first day and further decreased in next 24 h. The seeds of Sundre that remained non-germinated exhibited stable protein content.

The level of ATP in embryo (**Figure 2B**) sharply increased in the first 3 h after imbibition then declined reaching approximately the same level as in dry seeds after 24 h and declined further on the second day after imbibition. The level of ATP in non-germinated Sundre seeds was constant and similar to the level in dry seeds. The ratio of ATP and ADP (**Figure 2C**) also increased (from 2 to 6 in Harrington) in first 3 h, then declined to 4 after 10 h post imbibition, and increased again upon radicle protrusion with the same dynamics in both cultivars. The non-germinated seeds of Sundre kept almost the constant ATP/ADP ratio in embryo of approximately 2.5.

### NO Metabolism in Embryo of Germinating Seeds

**FIGURE 3:**
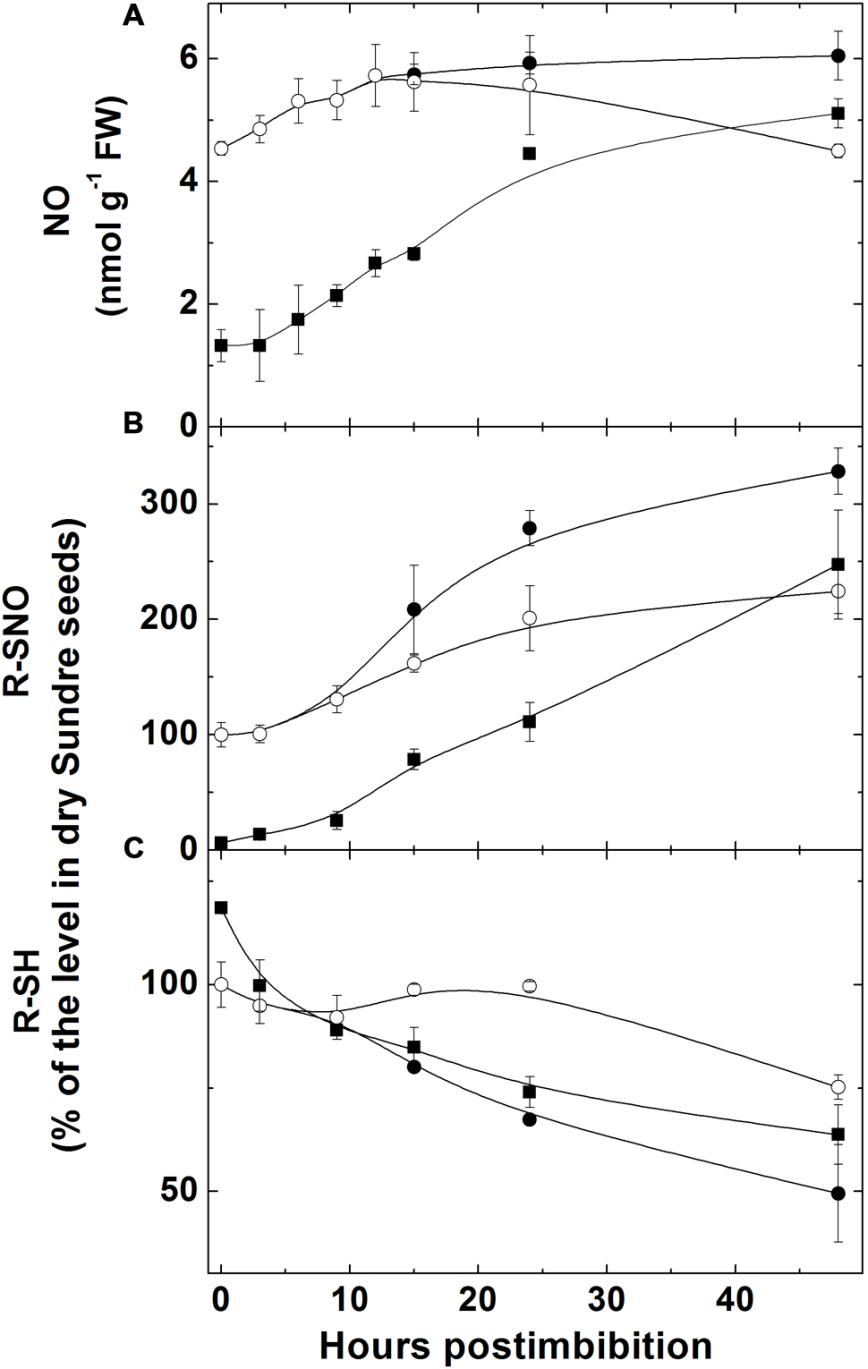
**Changes in NO level **(A)**, in the quantity of nitrosylated (-SNO) groups in proteins **(B)**, and the quantity of sulfhydryl groups in proteins **(C)** in embryo of two barley cultivars during germination.** The symbols are the same as in **Figure 2**.

**FIGURE 4:**
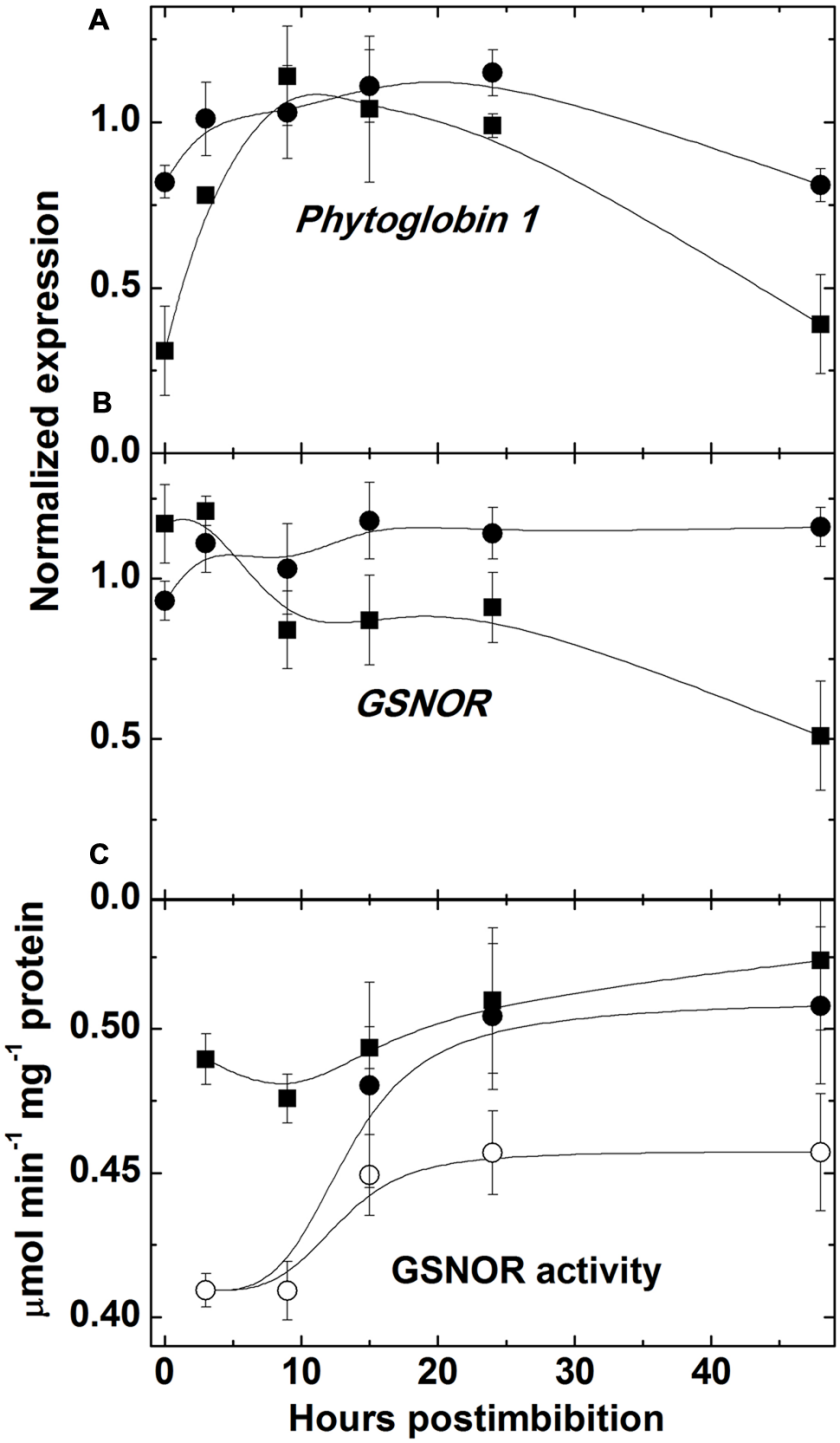
**Expression of the genes encoding class 1 phytoglobin **(A)** and GSNO reductase **(B)** and GSNO reductase activity **(C)** in embryo of two barley cultivars during germination.** The symbols are the same as in **Figure 2**.

The level of NO, measured with use of hemoglobin, increased (sharply in Harrington) in embryo in the first day starting from imbibition and then the increase became slower (**Figure 3A**). There was no increase in embryo of seeds of Sundre cultivar that remained non-germinated (even some decrease was observed). The increase of nitrosylation of SH-groups of proteins in embryo (**Figure 3B**) was continuously increasing after imbibition (only in the seeds remaining non-germinated this increase was slow). The level of free SH-groups in proteins decreased during germination remaining constant in non-germinated seeds (**Figure 3C**). The lower initial level of NO production in Harrington corresponded to lower rate of nitrosylation in the first hours after germination. The genes encoding the proteins participating in NO metabolism (class 1 phytoglobin and GSNOR) were expressed in germinating seeds (**Figures 4A,B**). While the expression of GSNOR was practically constant during 48 h post imbibition (slightly decreasing in Sundre), the expression of phytoglobin I gene increased in the first hours after imbibition, and started to decline on the second day post imbibition. There was no significant difference in expression of GSNOR and phytoglobin I genes between germinated and non-germinated seeds of the Sundre cultivar (not shown). The activity of GSNO-reductase increased during germination, more in Sundre than in Harrington, remaining at a lower level in non-germinated seeds (**Figure 4C**).

### H_2_O_2_ and $O_{2}^{•-}$ Levels

**FIGURE 5:**
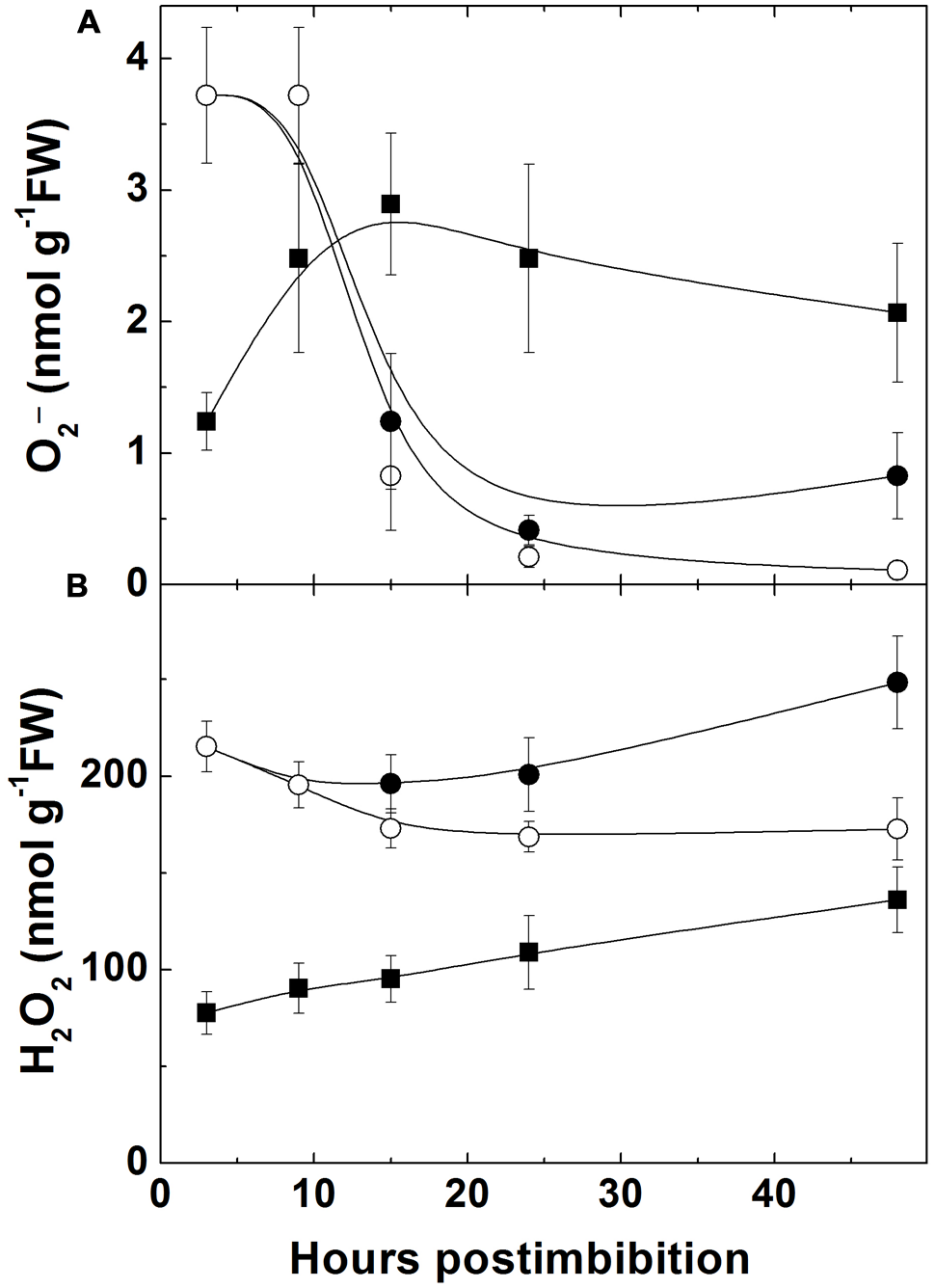
**Changes in the level of superoxide anion **(A)** and of hydrogen peroxide **(B)** in embryo of two barley cultivars during germination.** The symbols are the same as in **Figure 2**.

The level of superoxide was high in dry seeds of Sundre and low in Harrington. It decreased in Sundre upon imbibition and increased in Harrington (**Figure 5A**) being significantly higher in embryo after 10 h from imbibition. The content of H_2_O_2_ (**Figure 5B**) was higher in the Sundre cultivar remaining constant in non-germinated seeds, and having tendency of increase in germinating seeds in both cultivars, with a sharper increase in Harrington.

### Ascorbate and Glutathione Levels and Free SH-Groups

**FIGURE 6:**
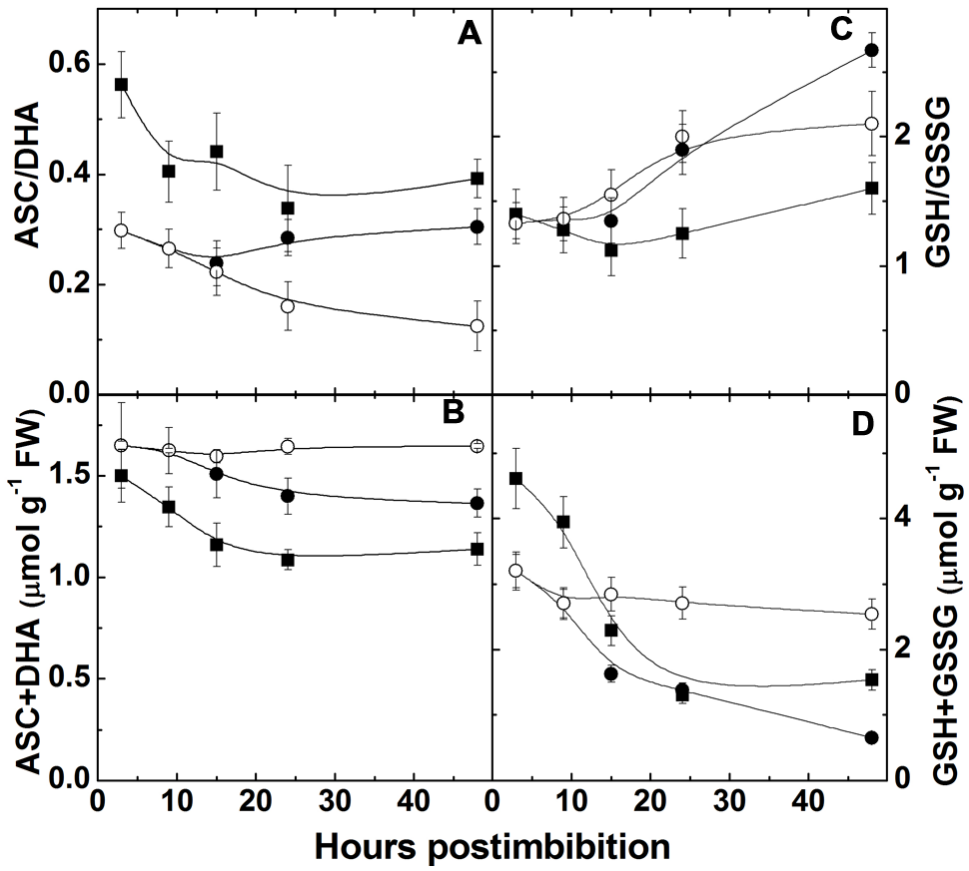
**Changes in the ratio ascorbate and dehydroascorbate **(A)**, total ascorbate pool **(B)**, the ratio of reduced and oxidized glutathione **(C)**, and total glutathione pool **(D)** in embryo of two barley cultivars during germination.** The symbols are the same as in **Figure 2**.

The ratio of ASC to DHA (**Figure 6A**) was initially higher in Harrington and then decreased, while the level of reduction of ascorbate pool in Sundre was significantly lower than in Harrington. The total ascorbate pool slightly decreased during the first day post imbibition in both cultivars, in dormant seeds it remained constant (**Figure 6B**). The level of reduction of glutathione decreased in Harrington in the first day, then increased; in Sundre the decrease was observed continuously from the start of imbibition (**Figure 6C**). These changes took place on the background of decrease in the total pool of glutathione (**Figure 6D**).

### Activities of Enzymes Involved in Oxidative Metabolism

**FIGURE 7:**
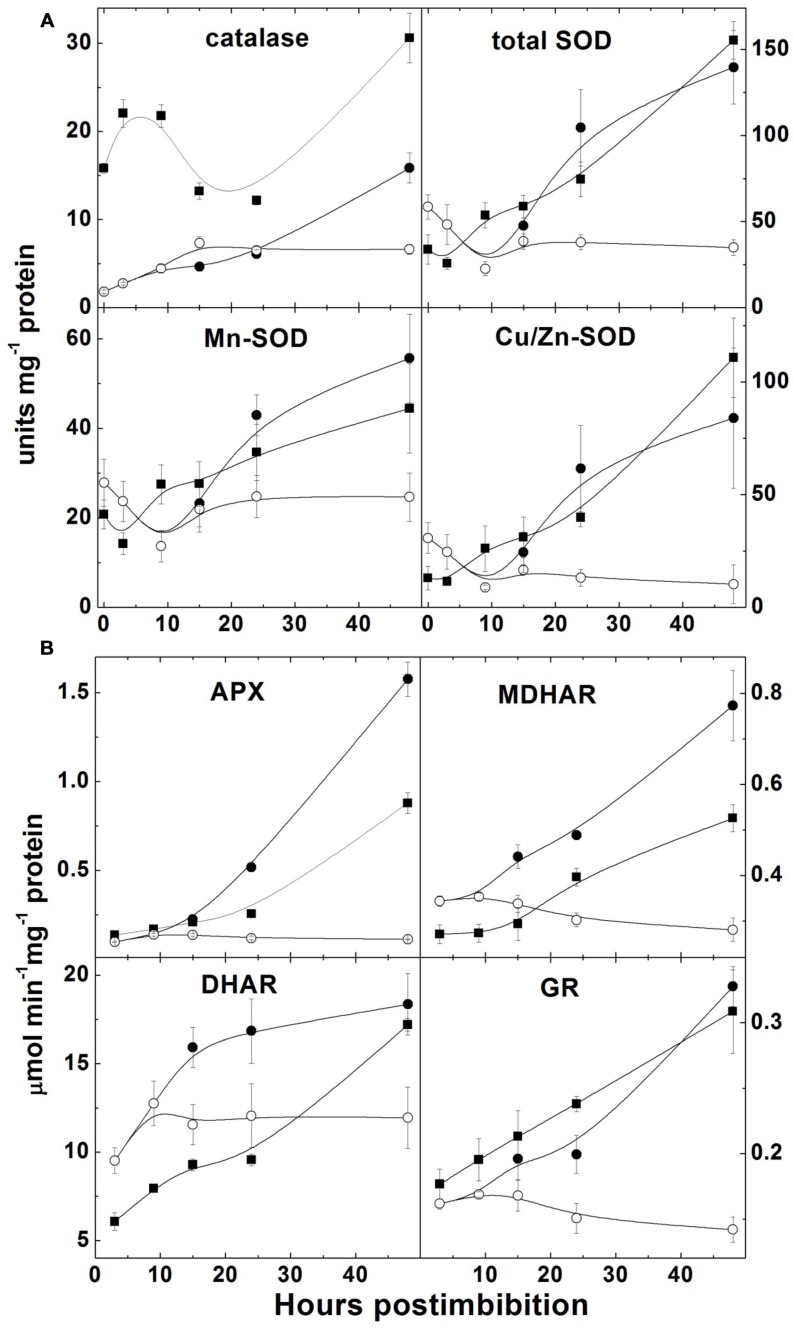
**Activities of the enzymes scavenging reactive oxygen species in barley embryos during germination.** **(A)** Activities of catalase and superoxide dismutase (total SOD, Mn-SOD, and Cu/Zn-SOD); **(B)** Activities of the enzymes of ascorbate-glutathione cycle (APX – ascorbate peroxidase, DHAR – dehydroascorbate reductase; MDHAR – monodehydroascorbate reductase; GR – glutathione reductase). Units of catalase are millimoles H_2_O_2_ min^–1^; units of SOD are 50% reduction of superoxide formation per minute. The symbols are the same as in **Figure 2**.

Catalase activity increased in Harrington in the first hours, then decreased and started to increase on the second day; in Sundre the activity was lower but the increase was continuous (there was no increase in non-germinated seeds; **Figure 7A**). SOD activity drastically increased after a period of low activity and some fluctuations in first 10–15 h (before radicle protrusion), these changes were similar for Cu/Zn-SOD and Mn-SOD and exhibited the same tendency in both cultivars. It did not show a change in the embryos of the seeds that remained non-germinated (**Figure 7A**).

All four enzymes of the ascorbate-glutathione cycle exhibited low initial activity and increased markedly during the first 48 h after imbibition (**Figure 7B**). Non-germinated seeds of Sundre cultivar kept low level of activity of all four enzymes.

**FIGURE 8:**
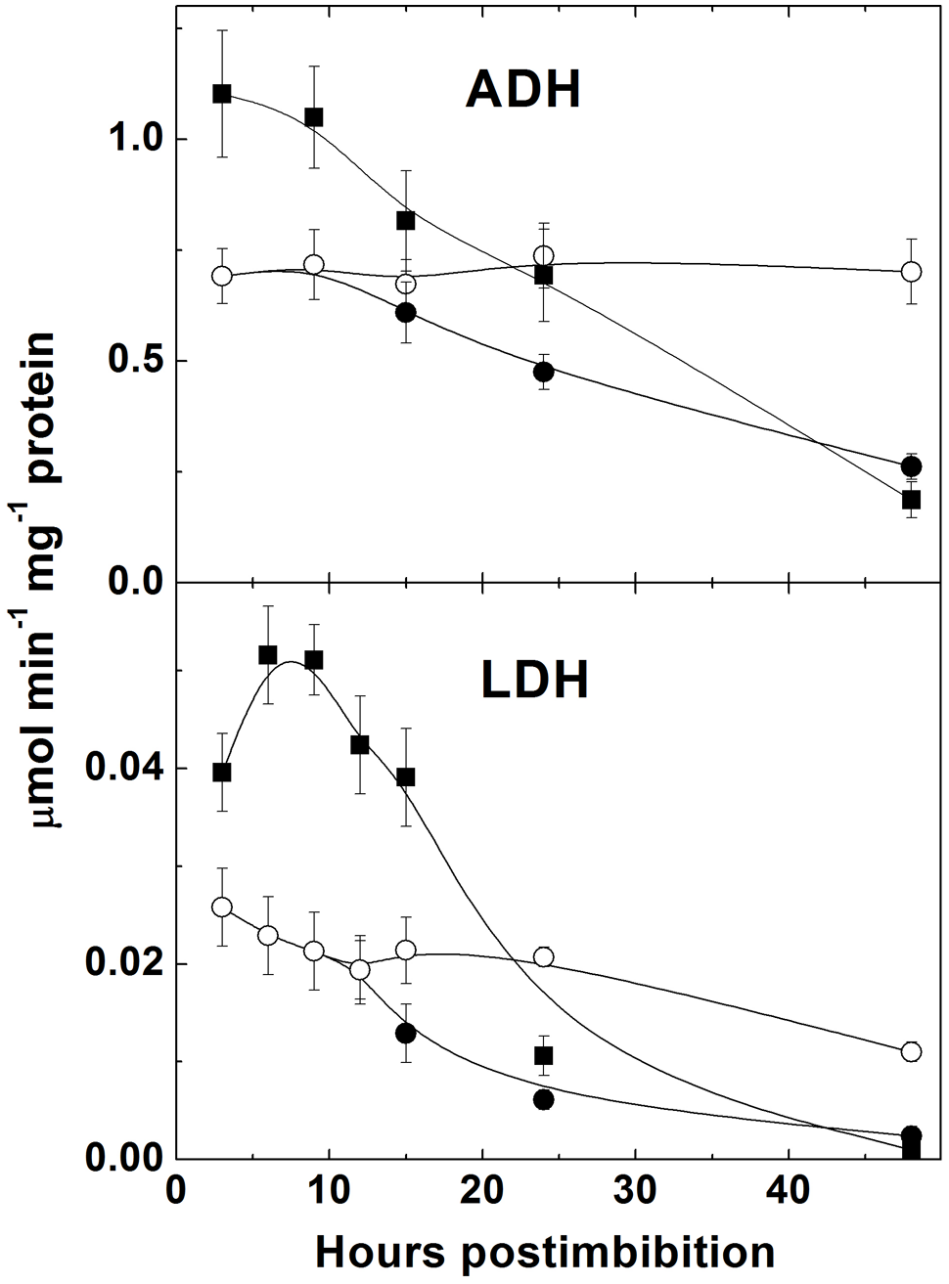
**Activities of fermentation enzymes alcohol dehydrogenase (ADH) and lactate dehydrogenase (LDH) in embryo of two barley cultivars during germination.** The symbols are the same as in **Figure 2**.

The activities of LDH and ADH were high in the first hours after imbibition and then decreased (**Figure 8**). They were significantly lower in more dormant Sundre cultivar and did not show change in the seeds that kept dormancy.

## Discussion

Seed germination is a process that starts from imbibition and results in radicle protrusion initiating seedling development. This process corresponds to the two stages described by Bewley and Black (1994), in the first of which rapid depletion of oxygen takes place and in the second the conditions inside seed are close to anaerobic. After radicle protrusion, the third stage starts in which oxygen concentrations gradually return to aerobic, active mobilization of storage reserves takes place and the seedling begins to develop. In barley seeds, radicle protrusion occurs within the first 24 h after imbibition. In our study the first radicles appeared between 12 and 15 h, and by 24 h all non-dormant seeds developed radicles (**Figure 1**). The separation between the first and second phases is conventional but we assume that by 3–5 h from imbibition most of oxygen is depleted and then the seeds exist in mostly anaerobic conditions until radicle protrusion.

Germination is characterized by the gradual utilization of storage reserves and a buildup of ATP, which is used for biosynthetic processes (**Figure 2**). In the seeds remaining dormant the protein level remains without change and the ATP/ADP ratio is constant being near 2.5. The first peak of ATP level is observed at 3 h post imbibition, which probably corresponds to the peak of aerobic metabolism before oxygen becomes depleted. Higher ATP levels in the non-dormant cultivar might reflect higher respiration capacity which in turn could result in higher production of superoxide in the side reactions of mitochondrial metabolism (Møller, 2001). After reaching its peak ATP level sharply declines, reflecting the development of anaerobic conditions, however, the observed decline in the ATP/ADP ratio is not strikingly pronounced and the ATP/ADP ratio, reaching the value 6 in Harrington at 3 h, declines only to the value slightly higher than 4 at 10 h. This indicates that even under highly hypoxic conditions there exist the mechanisms supporting ATP synthesis. Besides fermentation, NO turnover can be a contributor to ATP synthesis in these conditions (Stoimenova et al., 2007). Higher activities of fermentation enzymes (ADH and LDH) were indeed observed in germinating barley seeds in the first hours post imbibition, and the cultivar Harrington having higher ATP/ADP ratio at the first day is characterized by higher levels of ADH and LDH (**Figure 8**). It is also characterized by a sharper increase in phytoglobin expression (**Figure 4A**), which may also contribute to a buildup of ATP via the phytoglobin-nitric oxide cycle (Stoimenova et al., 2007; Gupta and Igamberdiev, 2011). Phytoglobin gene expression precedes the expression of phytoglobin protein which was detected in barley grains in the earlier study (Duff et al., 1998).

Indeed, the production of NO was shown to increase in barley seeds starting immediately from the onset of imbibition (**Figure 5A**). NO levels were initially higher in Sundre (more dormant cultivar) remaining stable in the seeds that kept dormancy, while in Harrington the increase in NO production was faster. The increase was most pronounced at the initial stages of germination, when the seeds become more anaerobic. This corresponds to the mechanism of reductive pathway of NO formation (via nitrite reduction at high redox level) facilitated by transition to anaerobic conditions (Dordas et al., 2003). The oxidative pathway of NO production is considered to be less active in plants and may be absent in germinating seeds (Igamberdiev et al., 2014). It is known that the development of anaerobic conditions in seeds results in a switch from oxygenic respiration to fermentation. An alternative to fermentation pathway could be NO turnover in the phytoglobin-nitric oxide cycle, in which NO is produced by reduction of nitrite and scavenged with formation of nitrate, the reaction catalyzed by a system involving the class 1 phytoglobin (Igamberdiev and Hill, 2004; Gupta and Igamberdiev, 2011). Both pathways operate during seed germination, and the activities of ADH and LDH (**Figure 8**) and the expression of phytoglobin (**Figure 4A**) exhibit cultivar-specific patterns.

The production of NO leads to nitrosylation of SH groups in proteins, peptides, free cysteine and its derivatives. The redox and energy level of germinating seeds is controlled via the balance of NO producing and scavenging mechanisms (Bykova et al., 2015). The most important scavenging mechanism for NO is its oxygenation to nitrate with participation of class 1 phytoglobin, which is hypoxically induced (Igamberdiev and Hill, 2004). While NO can be scavenged via phytoglobin-mediated mechanism, the most important RNS species, *S*-nitrosoglutathione is scavenged by GSNOR, which is the class III alcohol dehydrogenase having also the activity of formaldehyde dehydrogenase (Corpas et al., 2013). The activity of this enzyme was high already in dry seeds and it remained higher in the cultivar with high germination rate (Harrington; **Figure 4C**). The transcript expression of this enzyme (**Figure 4B**) was relatively constant from the onset of germination; therefore the observed changes in activity could be attributed to the posttranscriptional regulation. On the contrary, phytoglobin expression started to increase from the beginning of germination, remained high between 3 and 24 h and then declined; the changes were more expressed in the cultivar Harrington (**Figure 4A**). This is in agreement with the reported dependence of phytoglobin expression on the hypoxic conditions (Igamberdiev and Hill, 2004).

While we did not measure the concentration of *S*-nitrosoglutathione in this study, the decline in total glutathione (GSH + GSSG) pool (**Figure 6D**) can be explained, in particular, by its intensive nitrosylation. Other modifications of thiol groups caused by the increase in ROS formation may be responsible for the observed changes. Nitrosylation and other modifications also explain the decrease of free SH-groups in proteins (**Figure 3C**), which is supported by the observed increase of nitrosylated (-SNO) groups (**Figure 3B**), which follows the pattern of NO production with some delay (the increase in nitrosylation becomes more pronounced after 10 h post imbibition). The production of RNS (NO and nitrosylated compounds) during seed germination was suggested to be connected with dormancy alleviation (Bethke et al., 2004) but later it was suggested that inhibition of respiration by NO triggers ROS formation and that results in dormancy alleviation (reviewed in Bykova et al., 2015). We observed that in the seeds remaining dormant the increase in NO did not take place and the nitrosylation process did not actively develop (**Figure 3**).

In the course of germination upon the development of anaerobic conditions we observe oxidation of ascorbate and glutathione pools, while after radicle protrusion (at 15 h post imbibition) the ratios start to increase (**Figures 6A,C**). The glutathione pool and its reduction is the indicator of the overall cellular environment and important in control of morphogenetic processes (Mitrovic et al., 2012). Schafer and Buettner (2001) suggested that the resulting action of the glutathione redox couple triggers metabolic events and determines morphogenesis. GSH stimulates *Arabidopsis* root growth (Sanchez-Fernandez et al., 1997) favoring cell division and proliferation, while the GSSG content is related to differentiation (Schafer and Buettner, 2001; Stasolla et al., 2004). The changes of glutathione pool and its reduction level observed during germination are much more pronounced than of the ascorbate pool (**Figure 6**).

The importance of glutathione pool size and its reduction for determination of cell division, growth, and even apoptosis was mentioned in many works (reviewed in Noctor et al., 2012). The quiescent parts of plants such as root quiescent center, and cells in organs such as seeds maintain a highly oxidized intracellular state, in particular reflected in low content of reduction of the glutathione pool (Kranner et al., 2006), and the increase in the total cellular GSH pool is essential for the cells to progress to cell division (Diaz-Vivancos et al., 2010). Glutathione synthesis is required for pollen germination and pollen tube growth (Zechmann et al., 2011). The direct effect of glutathione has been observed in relation to its potential in meristematic cells; however, total tissue concentrations could give indirect indication of the direction of the morphogenetic process. Our study shows that the total pools of glutathione decreased after imbibition while its reduction increased after radicle protrusion. The redox state of glutathione can be tightly controlled by NO production and scavenging via phytoglobin and GSNOR mechanism. Thus NO can be an important regulator of dormancy release and tissue differentiation during seed germination.

The cross-talk between NO and ROS formation is important even during seed germination. The production of ROS occurs actively upon imbibition even in the period of oxygen depletion (**Figure 5**). Antioxidant defense mechanisms protect seeds during the dormancy stage and prevent their germination, while the break of dormancy is achieved in conditions when ROS production breaks these control mechanisms (Bykova et al., 2011a,b; Bykova and Rampitsch, 2013). Some data relate dormancy alleviation with the accumulation of H_2_O_2_ (Oracz et al., 2007; Bailly et al., 2008), however, the role of superoxide anion was also considered. It has been demonstrated that germination in *Arabidopsis* is mediated by accumulation of $O_{2}^{•-}$ and H_2_O_2_ in the radicle (Leymarie et al., 2012). In sunflower (*Helianthus annuus* L.) seeds, germination was shown to be associated with a marked increase in hydrogen peroxide and superoxide anion generation in the embryonic axes resulting from an inhibition of catalase and SOD and from activation of NADPH oxidase (Oracz et al., 2009). The role of NO in germination was also claimed (Bethke et al., 2004), and its interaction with ROS results in the balance of both ROS and RNS compounds that provides the conditions for germination.

The increase of superoxide levels in the non-dormant cultivar Harrington and its decrease in more dormant Sundre was observed during the first day, while the levels of H_2_O_2_ were lower in the seeds of the non-dormant cultivar (**Figure 5**). This might indicate that the main ROS player in alleviating dormancy in barley is indeed superoxide anion. On the other hand, some role of H_2_O_2_ in promotion of germination follows from the fact that in the seeds remaining non-germinated its level is lower than in the germinating seeds of the Sundre cultivar. The differences between the levels of superoxide and hydrogen peroxide could be related to the activities of corresponding scavenging enzymes (**Figure 7A**). However, before radicle protrusion the activities of SOD and of the enzymes of ascorbate-glutathione cycle were low and they started to increase drastically mostly on the second day of germination. The higher activity of catalase in Harrington may explain the lower H_2_O_2_ level at the onset of germination. Cakmak et al. (1993) also reported that during imbibition of wheat seeds the increase in activities of H_2_O_2_ scavenging enzymes is much more pronounced than the increase in SOD. In our study the increase in SOD (both the mitochondrial Mn-SOD and cytosolic Cu/Zn-SOD) was observed mainly on the second day of germination, i.e., after radical protrusion and switch to aerobic metabolism. The role of ascorbate-glutathione cycle is important not only for scavenging of H_2_O_2_ but also for establishing redox potentials of ascorbate and glutathione, which are the best indicators of the overall cellular environment and important in control of morphogenetic processes (Mitrovic et al., 2012; Talukdar, 2012).

## Conclusion

The production of NO and ROS during germination and their cross-talk are the important events that take place during germination of barley seeds. The class 1 phytoglobin, GSNOR and interaction with ROS play a role in scavenging and turnover of NO and nitrosylated compounds during germination. Although the observed differences between the two cultivars may be not necessarily be attributed to the dormancy levels, it becomes evident that the turnover of NO contributes to the maintenance of redox and energy balance of germinating seeds and may be an important player in alleviation of dormancy.

## Author Contributions

ZM performed all experiments, participated in discussion of results, contributed to writing the manuscript. FM planned and supervised the experiments on gene expression, analyzed results, contributed to writing the manuscript. NB planned and supervised the experiments on measurement of enzymes and metabolites, analyzed results, contributed to writing the manuscript. AI supervised the design, execution and interpretation of the experiments, prepared the manuscript.

## Conflict of Interest Statement

The authors declare that the research was conducted in the absence of any commercial or financial relationships that could be construed as a potential conflict of interest.
